# Association between serum zinc levels and lung cancer: a meta-analysis of observational studies

**DOI:** 10.1186/s12957-019-1617-5

**Published:** 2019-05-06

**Authors:** Ying Wang, Zhengyi Sun, Aipeng Li, Yongsheng Zhang

**Affiliations:** 1 Institute of Pediatrics, The First Hospital of Jilin University, Changchun, 130021 Jilin Province China; 2 Department of Ophthalmology, The First Hospital of Jilin University, Changchun, 130021 Jilin Province China; 3 Center for Reproductive Medicine and Center for Prenatal Diagnosis, The First Hospital of Jilin University, No. 71, Xinmin Street, Changchun, 130021 Jilin Province China

**Keywords:** Serum zinc or serum zinc level, Lung cancer, Meta-analysis

## Abstract

**Background:**

Inconsistent results according to numerous studies that had investigated the association between serum zinc levels and lung cancer risk were reported. The aim of this study was to explore whether serum zinc levels were lower in lung cancer patients than that in controls.

**Methods:**

We systematically retrieved the databases of PubMed, Wanfang, Cochrane, ScienceDirect website, CNKI, and SinoMed databases for comprehensive relevant studies published before December 2018 and conducted a meta-analysis. Standard mean differences (SMD) were pooled using a random effects model.

**Results:**

Thirty-two articles were eligible to investigate the correlation between serum zinc levels and lung cancer risk, involving 2894 cases and 9419 controls. The pooled results showed sufficient evidence approving the association between serum zinc levels and lung cancer risk. And the serum zinc levels in lung cancer were significantly lower than that in controls (summary SMD = − 0.88, 95% confidence interval (CI) = − 0.94, − 0.82). Meanwhile, consistent results were obtained both in European populations and Asian populations. No publication bias was detected in our analysis.

**Conclusions:**

The present meta-analysis suggested that serum zinc levels were significantly lower in lung cancer patients than that in controls.

## Introduction

Cancer is a crucial health problem on a global scale that has become one of the primary causes of death. The increasing trend in cancer globally could be slowed and reversed if preventive measures could provide the feasible approach [1]. As we all know, smoking had been as the well-known role in the development of lung cancer [2]. Previous studies had confirmed that some external environment exposure [3, 4], dietary factors [5, 6], and physical activities [7] could affect the risk of lung cancer. However, some trace element concentrations, such as zinc, copper, and so on, could also influence the development of lung cancer. A recent meta-analysis had been performed to explore the relationship about serum copper levels in lung cancer [8]. Copper and zinc are closely related trace elements. Zinc is used for the growth of cells and is also useful in maintaining the integrity of the cell membrane. Therefore, cancer cells may consume zinc in the circulation to maintain cancer growth and maintain its membrane integrity [9]. However, there has not been an article attempting to summarize the results for serum zinc levels on the risk of lung cancer. So far, numerous researchers have examined potential effects of serum zinc levels on lung cancer risk, but existing epidemiological data are inconsistent. Hence, we aimed to evaluate results from previous studies systematically and carefully by constructing a meta-analysis of observational studies to find whether serum zinc levels were lower in lung cancer patients than that in controls.

## Methods

This meta-analysis was designed and performed according to the guidelines of the preferred reporting items for systematic reviews and meta-analyses (PRISMA compliant) statement [10].

### Data sources and searches

A comprehensive, computerized literature search regarding the association between serum zinc levels and lung cancer risk was conducted in six databases (PubMed, Wanfang, Cochrane, ScienceDirect website, CNKI, and SinoMed databases), from their inception to December 2018. Combinations of the following keywords were used for the search: “zinc levels” OR “zinc concentration” OR “zinc” OR “trace element” in combination with “lung cancer” OR “lung tumor”. Moreover, we also scrutinized the references of retrieved publications to identify any studies that were potentially missed.

### Study selection criteria

To be eligible for our analysis, the studies had to meet the following criteria: (1) epidemiological studies; (2) the aim was to evaluate the associations between serum zinc levels and lung cancer risk; (3) the numbers, mean, and standard deviation (SD) of serum zinc levels for cases and control are available. It is noted that duplicated results may be published in more than one paper, so we selected the most recent or most informative paper in our analysis.

### Data extraction and quality assessment

Two of the authors extracted all data independently, complying with the selection criteria above. A standardized data collection protocol was as follows: the last name of the first author, publication year, study design, the location of the study conducted in, subject in cases and control, gender, range or mean age of cases, method of measurement for serum zinc, and mean and SD of serum zinc levels for cases and control.

### Statistical analysis

The strength of the association between serum zinc levels and lung cancer risk was measured by standard mean differences (SMD) and 95% confident interval (CI) by adopting random effects models that taking into account both within-study and between-study variations [11]. The analysis evaluated heterogeneity among researches via the Cochran’s *Q* test and *I*^2^ (inconsistency index) statistic [12]. Subgroup analysis based on study design and geographic location was conducted in this meta-analysis to explore possible heterogeneity and to analyze whether there was a correlation in some subgroups. The meta-regression analysis was also performed to examine the possible heterogeneity [13]. Furthermore, sensitivity analysis was done to estimate the stability of the results by removing each study from the analysis, one at a time, which can evaluate the influence of a single comparison on the overall risk estimate. We also adopted Begg’s funnel plots [14] and Egger’s linear regression test [15] to evaluate whether publication bias existed. All the statistical analyses involved were performed with STATA software. *P* values were two-sided and less than 0.05 was considered statistically significant.

## Results

### Literature search and study characteristics

**Fig. 1:**
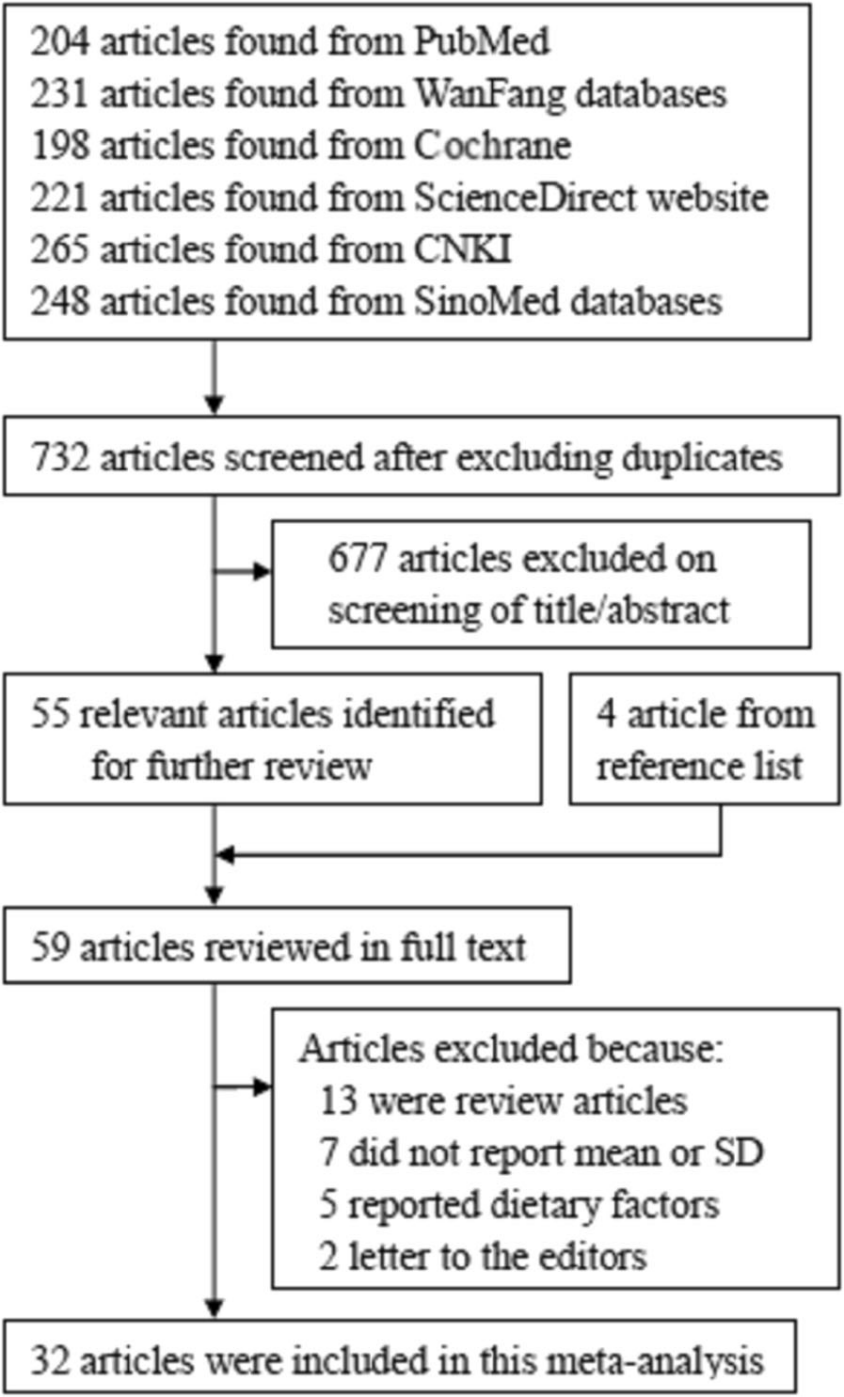
Flow diagram of the literature search

The specific step of searching and selecting relevant articles was summarized in Fig. 1. To sum up, we retrieved 204 articles from PubMed, 231 articles from Wanfang databases, 198 articles from Cochrane, 221 articles from ScienceDirect website, 265 articles from CNKI, and 248 articles from SinoMed databases. Fifty-nine articles were reviewed in full text. By evaluating the full text, 27 articles were further excluded owing to listed reasons below: review articles (*n* = 13), not reported mean or SD (*n* = 7), reported dietary factors (*n* = 5), and letter to the editor (*n* = 2). Ultimately, 32 articles [16–47] met the inclusion criteria. Three studies came from Europe and the remaining 29 studies were from Asia. The characteristics of the included studies are shown in Table 1.

**Table 1:** Characteristics of studies between serum zinc levels and lung cancer risk

Study, year	Country	Study type	Age	Lung cancer cases		Controls		Methods of measured zinc
				*n*	Zinc: mean ± SD	*n*	Zinc: mean ± SD	
Sun et al., 1991	China	Case-control	30–75	104	M = 0.945 ± 0.303(μg/mL) F = 0.970 ± 0.229 (μg/mL)	252	M = 0.945 ± 0.23(μg/mL) F = 1.08 ± 0.197 (μg/mL)	Atomic absorption spectrophotometer measurements (IL-951, USA)
Cobanoglu et al., 2010	Turkey	Case-control	54 ± 8.29	30	0.539 ± 0.225 (μg/dL)	20	2.051 ± 0.298 (μg/dL)	UNICAM-929 spectrophotometer (Unicam Ltd, York Street, Cambridge, UK)
Diez et al., 1989	Spain	Case-control	60 ± 7	64	0.627 ± 0.136 (μg/mL)	100	0.733 ± 0.173 (μg/mL)	Perkin-Elmer 5.000 atomic absorption spectrophotometer
Jin et al., 2011	China	Case-control	34.9 ± 21.3	154	0.673 ± 0.47 (μg/mL)	154	1.27 ± 0.442 (μg/mL)	Atomic absorption spectrophotometer (Wako Pure Chemical Industries, Osaka, Japan)
Oyama et al., 1994	Japan	Case-control	26–83	109	0.853 ± 0.279 (μg/mL)	53	0.873 ± 0.324 (μg/mL)	Atomic absorption spectrophotometry (Wako Pure Chemical Industries, Osaka, Japan)
Zablocka-Slowinska et al., 2018	Poland	Case-control	50–70	44	0.86 ± 0.215 (μg/L)	44	0.99 ± 0.263 (μg/L)	Atomic absorption spectrometry
Zowczak et al., 2001	Poland	Case-control	42–87	14	12 ± 2 (μmol/L)	18	13.8 ± 2 (μmol/L)	Flame atomic absorption spectrometry using Perkin ELmer spectrometer
Feng et al., 2006	China	Observation study	18–82	13	17.72 ± 2.55 (μmol/L)	36	17.57 ± 2.74 (μmol/L)	Flame atomic absorption spectrometry
Zhang et al., 1997	China	Case-control	25–80	64	0.788 ± 0.171 (μg/mL)	31	0.91 ± 0.374 (μg/mL)	Atomic absorption spectrophotometer measurements
Jin et al., 2001	China	Case-control	45–70	40	11.1 ± 3.73 (μmol/L)	46	13.4 ± 2.5 (μmol/L)	Atomic absorption spectrophotometer measurements
Zhang et al., 1994	China	Case-control	59 ± 9	40	16.98 ± 3.06 (μmol/L)	24	20.96 ± 3.67 (μmol/L)	Atomic absorption spectrophotometer measurements
Xu et al., 1993	China	Case-control	56 ± 7.5	42	11.45 ± 2.08 (μmol/L)	40	13.78 ± 2.12 (μmol/L)	Atomic absorption spectrophotometer measurements
Zhou et al., 1995	China	Case-control	39–69	186	0.702 ± 0.182 (μg/mL)	150	0.976 ± 0.102 (μg/mL)	Atomic absorption spectrophotometer measurements
Chen et al., 1994	China	Case-control	37–72	58	27.4 ± 4.8 (mol/L)	100	14.5 ± 3.8 (mol/L)	Atomic absorption spectrophotometer measurements (MFX-ID)
Luo et al., 1996	China	Case-control	40–70	35	10.25 ± 1.38 (μmol/L)	22	12.85 ± 4.28 (μmol/L)	Atomic Absorption Spectrophotometer measurements
Mo et al., 1995	China	Case-control	58.5	57	0.719 ± 0.159 (μg/mL)	46	0.955 ± 0.139 (μg/mL)	Atomic absorption spectrophotometer measurements
He et al., 1995	China	Case-control	34–72	143	19.481 ± 3.671(μmol/L)	50	21.436 ± 6.278(μmol/L)	Atomic absorption spectrophotometer measurements
Wei L et al., 2002	China	Case-control	22–76	79	0.832 ± 0.092 (μg/mL)	32	1.182 ± 0.018 (μg/mL)	Atomic absorption spectrophotometer measurements (American p-100)
Zhao et al., 1993	China	Case-control	43–62	46	11.17 ± 3.74 (μmol/L)	50	16.92 ± 5.6 (μmol/L)	Atomic absorption spectrophotometer measurements (BJKP-36)
He et al., 2011	China	Case-control	38–69	104	9.08 ± 1.44 (μmol/L)	122	16.44 ± 1.69 (μmol/L)	Atomic absorption spectrophotometer measurements
Chen et al., 1998	China	Case-control	47–72	43	11.74 ± 2.74 (μmol/L)	180	13 ± 1.83 (μmol/L)	Atomic absorption spectrophotometer measurements (Japan Shimadzu-A670)
Liang et al., 1992	China	Case-control	61	57	13.86 ± 5.56 (μmol/L)	80	21.81 ± 3.38 (μmol/L)	Atomic absorption spectrophotometer measurements (Chinese WFX-ID)
Huang et al., 1998	China	Case-control	25–65	136	11.933 ± 2.68 (μmol/L)	7101	19.808 ± 6.43(μmol/L)	Atomic absorption spectrophotometer measurements (Japan Shimadzu-AA670/C2H2)
Wang et al., 2003	China	Case-control	28–69	50	0.74 ± 0.18 (μg/L)	60	1.83 ± 1.44 (μg/L)	Atomic absorption spectrophotometer measurements
Cheng et al., 2011	China	Case-control	37–68	197	0.9 ± 0.3 (μmol/L)	93	1.12 ± 0.56 (μmol/L)	Atomic absorption spectrophotometer measurements
Xie et al., 2000	China	Case-control	35–68	64	70.28 ± 10.6 (μmol/L)	100	80.3 ± 20.7 (μmol/L)	Atomic absorption spectrophotometer measurements
Du et al., 1996	China	Case-control	22–73	73	13.1 ± 4 (μmol/L)	63	15.2 ± 3.5 (μmol/L)	Atomic absorption spectrophotometer measurements
Zhu et al., 1997	China	Case-control	NA	56	13.16 ± 1.71 (μmol/L)	118	15.46 ± 2.13 (μmol/L)	Atomic absorption spectrophotometer measurements (Perkin Elmer Zeeman/3030, USA)
Zhang et al., 2000	China	Case-control	25–77	310	0.812 ± 0.16 (μg/mL)	48	1.039 ± 0.154 (μg/mL)	Atomic absorption spectrophotometer measurements (Japan Shimadzu-180-80)
Hu et al., 2000	China	Case-control	36–77	56	0.797 ± 0.176 (μg/mL)	60	0.908 ± 0.022 (μg/mL)	Atomic Absorption Spectrophotometer measurements
Guo et al., 1994	China	Case-control	55.1	26	1.28 ± 0.94 (μg/mL)	26	0.8 ± 0.25 (μg/mL)	Atomic absorption spectrophotometer measurements (Varian Spectr AA-40p, USA)
Han et al., 1999	China	Case-control	4–77	400	0.93 ± 0.38 (μg/mL)	100	1.1 ± 0.36 (μg/mL)	Atomic absorption spectrophotometer measurements (American PE3030)

*SD* standard deviation, *NA* not available, *F* female, *M* male

### Serum zinc levels and lung cancer risk

**Fig. 2:**
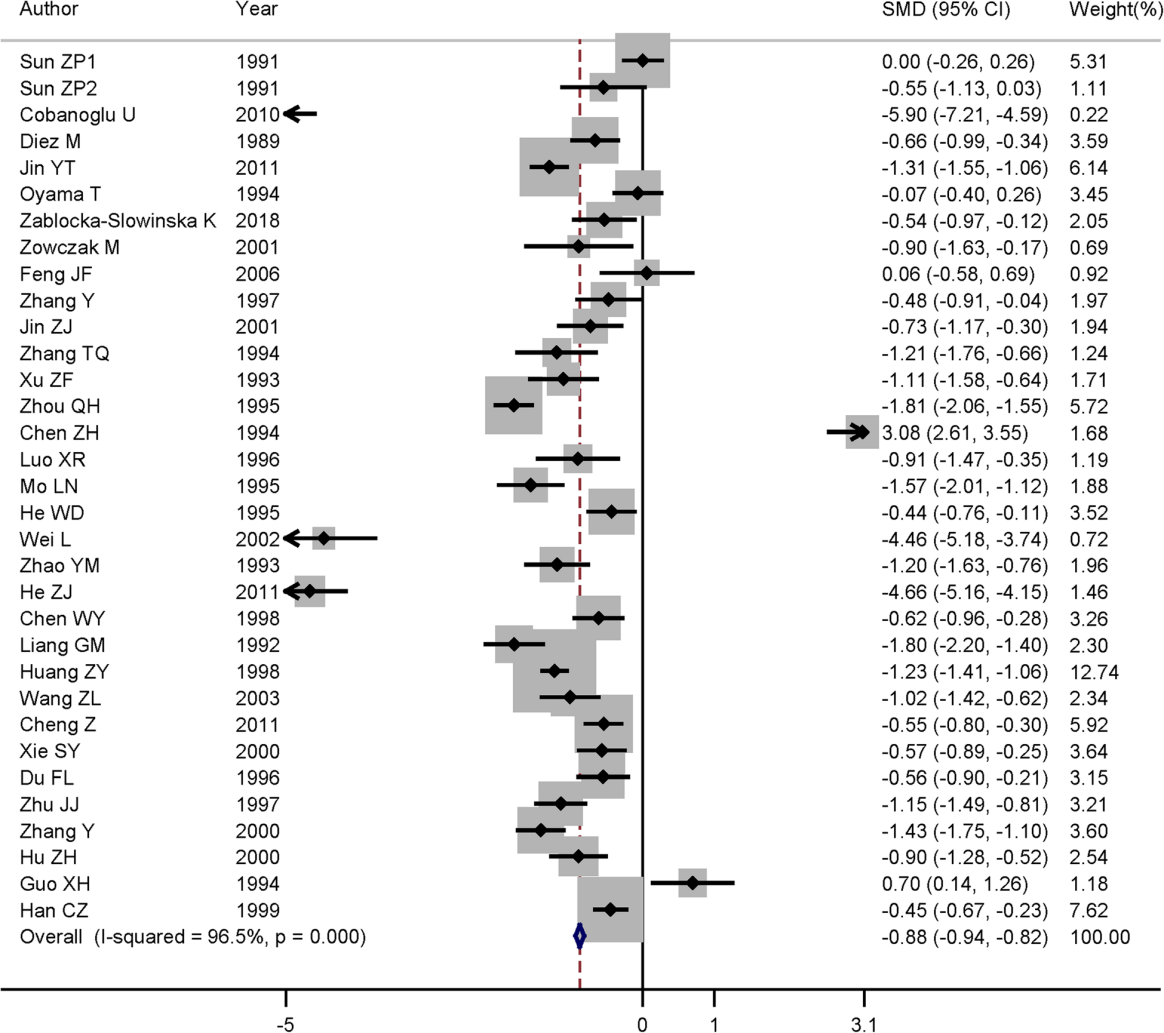
Forest plot of standard mean difference (SMD) with corresponding 95% confidence interval (CI) of studies about serum zinc levels and lung cancer risk

**Fig. 3:**
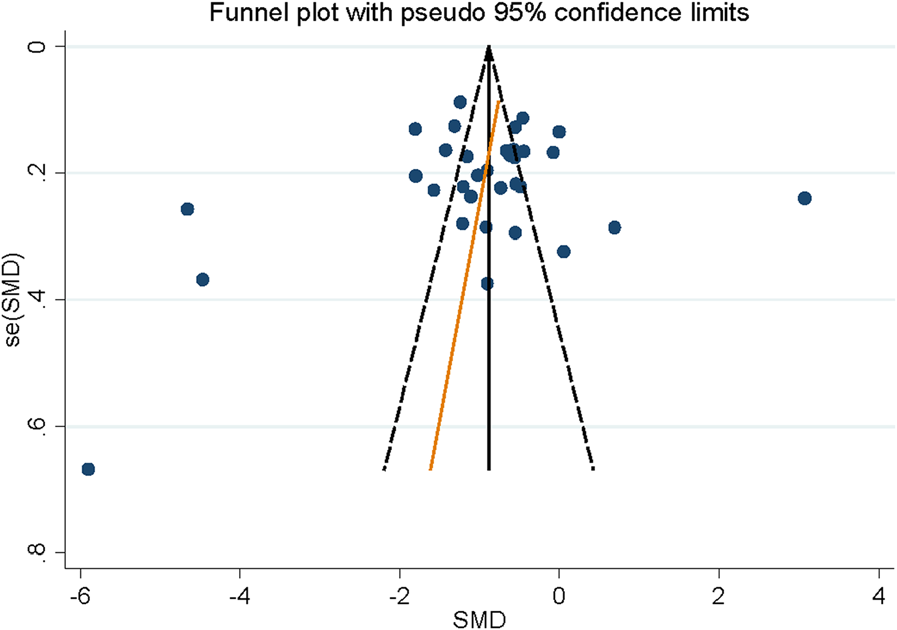
Funnel plot about serum zinc levels and lung cancer risk

In each study included in our analysis, 27 studies suggested that serum zinc levels were lower in lung cancer patients than that in controls, while four studies found a non-significant association between serum zinc levels and lung cancer. However, two studies obtained a positive association between serum zinc levels and lung cancer. Figure 2 has demonstrated the investigation results of the association between serum zinc levels and lung cancer in all the articles, as serum zinc levels in lung cancer were significantly lower than controls (summary SMD = − 0.88, 95% CI = − 0.94, − 0.82, *Z* value = 28.32, *P* for *Z* test < 0.001). Extreme heterogeneity was present among the pooled results (*P* < 0.001, *I*^2^ = 96.5%). Based on Egger’s test (*P* = 0.548) and the Begg’s funnel plot (Fig. 3), there existed no publication bias.

### Stratified analysis

Subgroup analyses by study design and geographic location were conducted to further examine serum zinc levels and lung cancer risk. We found lower serum zinc levels in lung cancer patients than that in controls both in European populations (summary SMD = − 0.65, 95% CI = − 0.89, − 0.41, *Z* value = 5.25, *P* for *Z* test < 0.001, *I*^2^ = 0.0%) and Asian populations (summary SMD = − 0.90, 95% CI = − 0.96, − 0.83, *Z* value = 27.90, *P* for *Z* test < 0.001, *I*^2^ = 96.8%). Thirty-one of the 32 studies were case-control studies, and the result was consistent with the overall result (summary SMD = − 0.89, 95%CI = − 0.95, − 0.83, *Z* value = 28.47, *P* for *Z* test < 0.001, *I*^2^ = 96.6%).

### Meta-regression analysis

Meta-regression analysis was evaluated in this article, indicating that no source of heterogeneity was observed in the association between serum zinc levels and lung cancer risk.

### Sensitivity analysis

Sensitivity analysis indicated that no evidence of overall result changing was observed when removed each study from the analysis, one at a time.

## Discussion

The meta-analysis was conducted to investigate the association between serum zinc levels and lung cancer. Findings from our report suggested that serum zinc levels in lung cancer cases were significantly lower than that in controls. Consistent results were found both in European populations and Asian populations.

Some previous studies had been published to explore serum element levels and lung cancer risk. Chen et al. performed a meta-analysis with 13 publications to assess the association between serum iron levels and lung cancer risk [48]. The authors concluded that serum iron levels had no effect on the risk of lung cancer. Song et al. found no significant association between serum magnesium levels and lung cancer risk when pooled 11 suitable papers [49]. However, Zhang et al. performed a meta-analysis using 33 articles to explore the association between serum copper levels and the risk of lung cancer [8]. Results from their study suggested that serum copper levels were higher in lung cancer than that in controls. Copper and zinc are closely related trace elements involved in cell proliferation, growth, gene expression, apoptosis, and other processes. These two trace elements are all necessary for the proper activity of superoxide dismutase due to their integral role as cofactors or ions stabilizing the molecular structure [50]. Zinc deficiency may have adverse events, especially on immune function [51]. Gómez et al. had studied the association of zinc and its role in lung cancer [52]. In general, zinc microenvironment may play a key role in oxidative stress, apoptosis, and/or cell signaling alterations which influence the behavior of malignant cancer cells [52], and this may play a role in preventing lung cancer.

Previous studies had significantly revealed that serum zinc had a protective effect on some cancers. Xie et al. conducted a meta-analysis about serum zinc levels and cervical cancer, indicating that serum zinc levels were lower in cervical cancer patients than controls [53]. In addition, Mao et al. found that bladder cancer patients had lower serum zinc levels compared with controls [54]. Moreover, a meta-analysis published by Zhao et al. suggested that serum zinc concentrations in prostate cancer patients were significantly lower than those in normal controls [55]. Our results were consistent with the abovementioned studies.

However, there are some limitations and potential bias that must be acknowledged in our meta-analysis. First, we only included papers published in English or Chinese, which may omit some other language paper. Furthermore, we only searched published articles, which may omit some unpublished articles or some meeting articles. These factors may yield between-study heterogeneity and publications bias in the overall pooled results. Second, although most method of measurement for serum zinc was using atomic absorption spectrophotometer, different methods of measurement as well as a different instrument could also cause between-study heterogeneity. Third, evidence of high heterogeneity was found, both in overall and subgroup analysis, in our analysis. However, we could not find the source of heterogeneity due to stratified analysis and meta->regression analysis concerning the relationship between serum zinc levels and lung cancer risk. Fourth, our meta-analysis included three articles from Europe and 29 articles from Asia, thus, further epidemiological studies are warranted in the future to assess the association between serum zinc levels and lung cancer risk.

## Conclusions

In summary, our meta-analysis, which included a large number of subjects of 32 articles, manifested that serum zinc levels were significantly lower in lung cancer patients than that in controls.

## Abbreviations

SMD: Standard mean differences
CI: Confidence interval
SD: Standard deviation
